# Contrasting ecological roles of non-native ungulates in a novel ecosystem

**DOI:** 10.1098/rsos.170151

**Published:** 2018-04-25

**Authors:** Ann Marie Gawel, Haldre S. Rogers, Ross H. Miller, Alexander M. Kerr

**Affiliations:** 1 Department of Ecology, Evolution, and Organismal Biology, Iowa State University, Ames, IA, USA; 2 College of Natural and Applied Sciences, University of Guam, UOG Station, Mangilao, GU, USA; 3 Marine Laboratory, University of Guam, UOG Station, Mangilao, GU, USA

**Keywords:** ungulates, novel ecosystems, seed dispersal, herbivory, Guam, Mariana Islands

## Abstract

Conservation has long focused on preserving or restoring pristine ecosystems. However, understanding and managing novel ecosystems has grown in importance as they outnumber pristine ecosystems worldwide. While non-native species may be neutral or detrimental in pristine ecosystems, it is possible that even notorious invaders could play beneficial or mixed roles in novel ecosystems. We examined the effects of two long-established non-native species—Philippine deer (*Rusa marianna*) and feral pigs (*Sus scrofa*)—in Guam, Micronesia, where native vertebrate frugivores are functionally absent leaving forests devoid of seed dispersers. We compared the roles of deer and pigs on seedling survival, seed dispersal and plant community structure in limestone karst forests. Deer, even at low abundances, had pronounced negative impacts on forest communities by decreasing seedling and vine abundance. By contrast, pigs showed no such relationship. Also, many viable seeds were found in pig scats, whereas few were found in deer scats, suggesting that pigs, but not deer, provide an ecosystem function—seed dispersal—that has been lost from Guam. Our study presents a discrepancy between the roles of two non-native species that are traditionally managed as a single entity, suggesting that ecological function, rather than identity as a non-native, may be more important to consider in managing novel systems.

## 1. Introduction

The extent of human influence is so pervasive that the Earth today is comprised mostly of novel ecosystems [1]—anthropogenically modified systems with species compositions and relative abundances that have not been previously observed [2]. Species introductions create and maintain novel ecosystems both by adding new species and by removing native ones [1]. Novel ecosystems typically still harbour many native species [3]; however, effective management of these systems is challenging due to the potentially new ecological roles of the remnant native and introduced species that comprise them.

Although the negative impacts of introduced species are extensive, some may also play beneficial roles [4]. Introduced species can be preferred candidates for restoring severely degraded habitats [5]. For example, non-native trees have been used in abandoned pastures where the native plants would not have originally facilitated the return of native plant communities [6]. Some introduced species may provide desirable ecological functions such as seed dispersal or food sources for native species [7]. The introduced Japanese white-eyes (*Zosterops japonica*) in Hawaii are seed dispersers for native plants that previously relied on now extinct or rare native birds [8]. Finally, invasive species may slow or reverse negative ecological effects from other anthropogenic impacts. Cascading ecological effects from overfishing in Cape Cod salt marshes are being reversed by green crabs (*Carcinas maenas*), which are normally considered a harmful invasive [9].

The negative effects of introduced deer and pigs have been well documented in ecosystems across the globe [10–13]. Deer alter forest structure by browsing on seedlings and saplings, and suppressing forest regeneration [14]. Introduced deer in New Zealand had effects on understorey and forest composition that persisted even after the control of deer (*Cervus elaphus*) populations [15]. Similarly, pigs are known to affect regeneration and recruitment in a number of forest systems. For example, feral pigs (*Sus scrofa*) have a pronounced effect on regeneration in lowland forests of Malaysia, by direct predation on seeds and by soil-rooting [16,17]. Rooting kills or physically damages seedlings and alters soil properties [18,19].

The southernmost island of the Mariana Archipelago, Guam, as with many islands around the world, has had a long history of species introductions [20], making its forests prime examples of novel ecosystems, albeit with unique challenges. One of the world's most infamous invasive species is the brown treesnake (*Boiga irregularis*), which was unintentionally introduced to Guam on military cargo at the end of World War II [21]. It is responsible for the extinction of most of Guam's native birds between 1945 and 1985 [22,23], functionally leaving the island bereft of native vertebrate nectarivores, frugivores or insectivores [20]. While snakes are a relatively recent introduction, Philippine deer (*Rusa marianna*) (referred to as deer from here on) and feral pigs (*Sus scrofa*) (referred to as pigs from here on) have been established for centuries in Guam [24–26]. Deer were introduced to the wild in Guam in 1772 by Spanish Governor Mariano Tobias as game [27], while pigs in the forests of Guam are descended from livestock brought by Spanish colonizers in the 1660s, and subsequently mixed with other livestock throughout the centuries [24]. We have no evidence that wild boar have ever been introduced to Guam. The lone study estimating ungulate abundance on Guam used spotlight counts from multiple vehicles along abandoned runways on the Air Force Base (K. Knutson, S. Vogt 2003, unpublished data). They estimated 1.83 deer per hectare (95% confidence interval = 1.44–2.21) and feral pig densities of 0.38 pigs per hectare (95% confidence interval = 0.20–0.55), indicating that ungulates can reach high densities on Guam, particularly in this area with limited access for hunting.

Like ungulates in other systems, both deer and pigs are thought to have negative effects on plant communities in Guam [24–26,28] (K. Knutson, S. Vogt 2003, unpublished data). Deer density in Guam has been correlated with reduced seedling recruitment in some species of native trees [25,28]. Pigs in Guam, similar to pigs in other systems, alter habitats by rooting and wallowing [24], which can disrupt forest regeneration. However, the effects from deer and pigs are occurring within novel rather than pristine ecosystems; therefore, a more thorough examination of the role of each species within the larger ecological context is needed to make appropriate management decisions.

We investigated the ecological role of non-native ungulates in the novel ecosystems of Guam by examining the influence of non-native deer and pigs on seedling survival, seed dispersal and seedling abundance in limestone karst communities. First, we experimentally tested whether deer and pigs affect seedling survival of a specific subset of native and non-native plants. We also tested the capabilities of deer and pigs for dispersing seeds by germinating scats collected in the wild. Finally, we compared plant community characteristics (e.g. native and non-native seedling abundance, vine abundance) across a range of relative ungulate densities to assess whether the effects of ungulates as herbivores or dispersers are evident in the forest.

## 2. Methods

### 2.1. Study area

Guam (13.5° N, 144.8° E; 544 km^2^) is the largest and southernmost island of the Mariana Island Archipelago in the Western Pacific. The dominant forest type of Guam is limestone karst forest. Plant communities in these forests are growing on top of calcareous rock—the brittle, fossilized remains of ancient marine organisms. This karst is extremely porous and easily weathered by water, creating sharp features that hold very little topsoil [29,30]. It is extremely rugged, with small crevasses and holes throughout. While a variety of karst types exist in northern Guam, our seedling plot and transect sites all occurred on reef facies and detrital facies of what is classified as Mariana Limestone—that is, Plio-Pleistocene reef and lagoon that comprises 75% of Guam's karst formations [30].

Guam's karst forests were chosen as the focus of this study because they contain a larger variety of native and endemic tree species relative to other habitats, such as savannah or ravine forest [31,32]. We chose sites for this project that were considered native limestone karst forest in order to maintain similarities between sites and maximize the likelihood of discerning differences due to pig and deer abundance rather than other site characteristics like history of disturbance or species composition. Native trees still dominate these sites. However, the relative abundances of vegetation differ from early descriptions of Guam forests, suggesting that forests have changed over time [32,33]. This, combined with the presence of non-native plants, insects and mammals [20] and the absence of birds, provides an ideal setting for investigating roles of non-native species in a novel ecosystem.

### 2.2. Effect of ungulates on seedling survival

**Figure 1.**
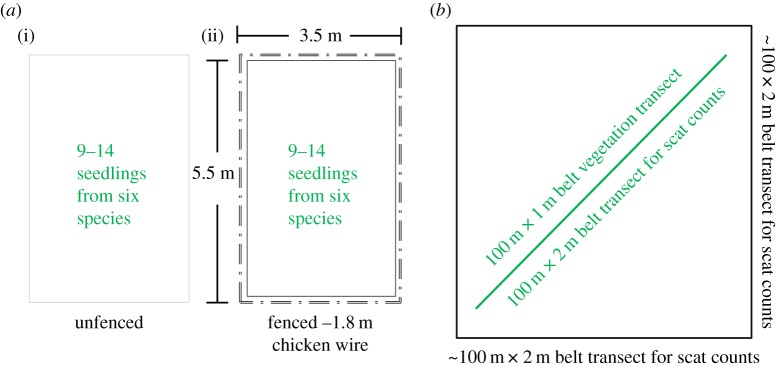
Diagram in (*a*) shows dimensions for adjacent fenced (no ungulates) and unfenced (ungulates) seedling plots constructed and planted at eight forest sites in Guam. Diagram in (*b*) illustrates the belt transects used to characterize vegetation and the larger belt transect where the surveyor walked the vegetation transect and the area around the vegetation transect to count scats within a 2 m width belt as a proxy for ungulate abundance. Transects were conducted at 14 forest sites in Guam.

To assess ungulate effects on seedling survival, we set up paired plots in eight selected karst forest sites in northern Guam. At each site, we erected a 1.8 m tall chicken-wire fence around one plot, and left the adjacent plot unfenced, allowing ungulate access. Each seedling plot covered an area of about 3.5 m × 5.5 m (figure 1). The fenced and unfenced plots were placed so that individual pairs had similar canopy cover, rockiness, slope and ground cover, as well as similar adult tree composition, density, heights and diameters (electronic supplementary material, tables S1–S3 and figure S3). While species composition of adult trees already present was almost impossible to match exactly, species composition often overlapped (electronic supplementary material, table S3). Using linear mixed-effect models and the lsmeans package for post hoc comparisons, we compared the fenced and unfenced treatments and found no significant differences in numbers of adult trees (*p* = 0.22), canopy cover (*p* = 0.92), average diameter at breast height (*p* = 0.57), and average height of adult trees (*p* = 0.98) between paired plots (electronic supplementary material, table S2 and figure S3). We avoided gaps, depressions in the substrate or any other landscape features that might have caused a difference between the paired plots*.*

We selected six species for this experiment encompassing a variety of native species—*Ochrosia oppositifolia* (synonym *Neisosperma oppositifolia*), *Aglaia mariannensis*, *Morinda citrifolia*, *Premna serratifolia* and *Psychotria mariana*—and one introduced tree species: *Carica papaya*. All are common components of Guam's limestone karst forests, although the non-native *C. papaya* tends to favour edges, and *P. mariana* is less common than the other species. For each species, we collected seeds from at least five trees and at least four different sites to minimize maternal effects and effects of local adaptation. The date of seed collection and subsequent out-planting was staggered by species due to differences in fruiting phenology. Seeds were planted under 60% shade cloth at a nursery and allowed to grow in these conditions until they had fully rooted and grown their first true leaves. At this point, the seedlings were transported to the exclosure sites for out-planting.

Seedlings of each species were out-planted in the control and treatment areas at each site on the same day. Seedlings were placed at least 0.3 m apart from each other, and at least 0.5 m away from the fences in fenced treatments. Seedlings were haphazardly placed within the seedling plot because they had to be planted around rocky karst structures and roots from neighbouring trees. Fourteen seedlings of each species were planted in each treatment at each site, except for *O. oppositifolia*, a tree with large fruits and seeds, which had only nine seedlings planted per treatment. The species (*C. papaya, M. citrifolia* and *O. oppositifolia*) planted during drier months (December through May) were watered every other day during the first few weeks following transplanting to ensure they successfully established. We monitored seedling mortality monthly, but we use the counts from the final survey conducted in July 2011, 15 months after the first species was transplanted and four months after the last, for the analysis.

As planting dates were staggered for each species and the duration of growing time varied by species, we first used model comparison to test whether the effect of treatment (ungulates or no ungulates) was better explained by the length of time in the ground or by species. After determining that duration of time in the ground was a poor predictor of treatment effects compared to species, we assessed treatment effects for each of the six species individually. Seedling survival was compared for each planted species between fenced and unfenced plots, using generalized linear mixed-effect models (lme4 package) in R [34,35]. Fencing was considered a fixed effect, whereas site was considered a random effect. A factor was considered to have a significant effect on seedling survival if its inclusion reduced Akaike information criterion scores, corrected for smaller sample sizes (AICc), by more than two in the corresponding model [36].

### 2.3. Germination from scats

We collected scats from deer and pigs from limestone karst forest sites to determine if either species dispersed viable seeds via endozoochory. We collected throughout the year, through both rainy and dry seasons from four sites in northern Guam. Scats were layered on top of a 50% perlite and 50% peat moss soil mix in germination trays. The trays were kept outdoors at a nursery under shade cloth. Deer pellets were admixed at the surface, pig scats were broken up and mixed at the surface. Trays were watered regularly. Seedlings were identified and counted after germination or after seedlings grew to a state where we were able to identify them. Because the nursery was open air, wind-dispersed species that germinated across all seedling trays, including adjacent experiments at the nursery, were not counted. We then compared the abundance of species germinating in scats with their abundances in the forest, using our vegetation transect data. Proportional abundance in the forest for each species was calculated by dividing the total count of adults of that species across all sites and dividing that by the total number of adult trees across all sites (total adult count of one species / total adult count across all species counted on vegetation transects). We counted only adult trees in calculations to represent potentially fruiting trees. We used a similar approach to calculate the proportional abundance of seedling species found in pig and deer scats: the total number of scats with a given species germinating from it, divided by the total number of either deer or pig scats collected.

### 2.4. Effects of ungulates on community composition

We surveyed the community composition of karst forest sites by using 100 m by 1 m belt transects at 14 different sites across northern Guam, where limestone karst dominates the forest types. All plants within these transects were identified to species and categorized by growth form (i.e. vines or trees) and as native or non-native. We categorized a plant as a seedling if it appeared to be within its first year of growth; for woody species, indicators included the lack of a woody stem, or a height less than 0.5 m.

Scats from pigs and deer were counted along the vegetation transects described above and along four 100 m long by 2 m wide belt transects encircling the vegetation transects (figure 1). The precise length was recorded using GPS, with total area surveyed amounting to approximately 1000 m^2^. However, because transect lengths used to count scats differed slightly from site to site, scat abundances used in analysis were number of scats per 100 m^2^. Actual ungulate densities in limestone forest on Guam are unknown and we do not attempt to measure ungulate abundance here. Although scat counts do not give exact population abundance, they can be used as an index to compare abundance of ungulates among sites [37]. We used scat as an indicator of ungulate abundance because other signs, such as animal tracks, are rarely visible in karst forest terrain and the detectability of other sign such as trails and browsing sign varies widely even in similar habitats [25].

We used linear regressions to determine whether ungulate scat abundance covaried with forest characteristics measured on vegetation transects. Deer scat abundance and pig scat abundance were considered in separate models, although a model including both provided qualitatively similar results (electronic supplementary material, tables S4–S7). The forest characteristics included as dependent variables were total seedling abundance, native seedling abundance, non-native seedling abundance and vine abundance. For each correlation, we report *r*^2^-values.

## 3. Results

### 3.1. Effects of ungulates on seedling survival

In a full model with data from all six species, the effect of treatment did not depend on growing time (the best-fit model did not include a time by treatment interaction; table 1), but the effect of treatment differed by species. As we were interested in treatment effects on a species level, we then analysed treatment effects separately for each species.

**Table 1.** Generalized linear mixed-effect model comparison; main effects included length of time seedlings were in the ground, fenced or unfenced treatment and seedling species, as well as a treatment by species and a treatment by time interaction. The best-fit model did not include a treatment by time interaction, indicating that the effect of treatment did not depend on the growing time duration for seedling species.

model	no. parameters	AICc	ΔAICc
treatment, species, treatment : species	13	608.45	0
treatment, time, species, treatment : species, treatment : time	15	610.34	1.89
treatment, time, treatment : time	5	762.53	158.1

**Figure 2.**
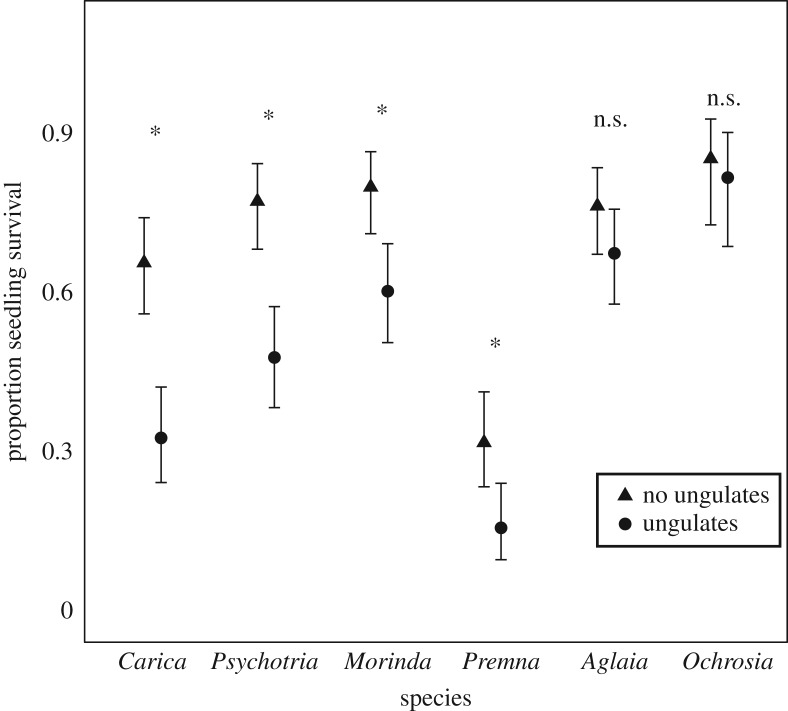
Proportion of seedlings that survived in fenced versus unfenced plots for six tree species. The asterisk (*) indicates that the best-fit model included treatment, indicating an effect of excluding ungulates on seedling survival.

For four species, *C. papaya*, *M. citrifolia*, *P. serratifolia* and *P. mariana*, fencing treatment contributed to the best-fit model explaining proportion alive (table 2 and figure 2), with higher survival of seedlings when protected from ungulates. For *A. mariannensis* and *O. oppositifolia*, AICc values were less than 2 between models including and not including treatment, indicating that these two species did not benefit from protection from ungulates. Almost all mortality observed in seedling plots was in the form of deer herbivory—as indicated by clipped leaves and stems—instead of disturbance due to uprooting by pigs.

**Table 2.** Generalized linear mixed-effect model comparisons; each of the six species was considered separately. For each species, a model including treatment (ungulates or no ungulates) was compared to a null model.

model	species	AICc	ΔAICc	AICc weight
with treatment	*Carica papaya*	121.13	0	1
null		155.81	34.68	0
with treatment	*Psychotria mariana*	78.39	0	1
null		100.51	22.12	0
with treatment	*Morinda citrifolia*	106.27	0	0.98
null		114.55	8.27	0.02
with treatment	*Premna serratifolia*	86.4	0	0.98
null		94.17	7.77	0.02
null	*Aglaia mariannensis*	79.36	0	0.56
with treatment		79.88	0.52	0.44
null	*Ochrosia oppositifolia*	46.22	0	0.8
with treatment		48.94	2.72	0.2

### 3.2. Germination from scats

**Figure 3.**
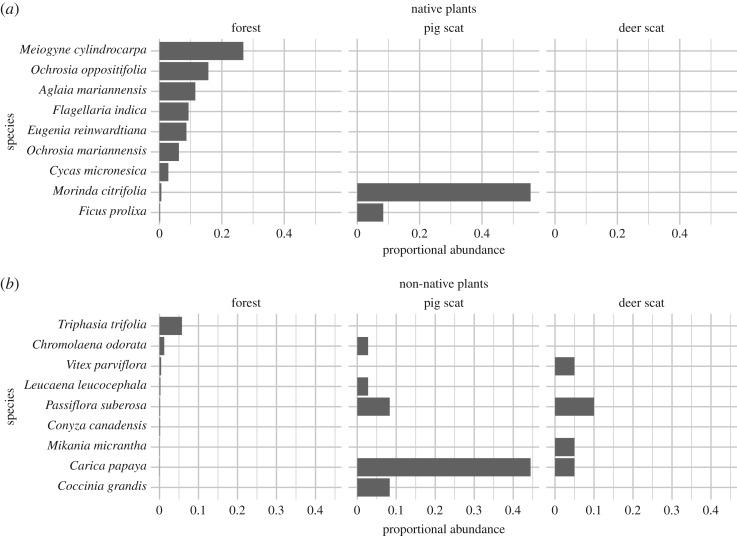
(*a*,*b*) The relative abundance of adult trees in the forest (left panel), and the relative abundance of seeds in pig (middle panel) and deer (right panel) scat. The most abundant tree species are shown at the top, with native species in the upper panel and non-native species in the lower panel. The top seven most abundant native and non-native species found during vegetation transects are included, in addition to any species found in pig or deer scat. Scat proportional abundance was calculated as the number of scats in which a species occurred out of the total number of scats collected.

Only four of the 20 deer scats collected produced seedlings (20%), and only 13 seedlings germinated, from four different species of non-native plants and two unknown species (table 3 and figure 3). Eight *Passiflora suberosa* seedlings germinated from one pellet group. In addition, one *C. papaya*, one *Vitex parviflora* and one *Mikania micrantha* seedling each emerged from separate pellet groups. The *M*. *micrantha* may have been ingested by the deer accidentally, as this species has wind-borne seeds lacking a fruit.

**Table 3.** List and counts of species germinated from deer scats (*n* = 20) and pig scats (*n* = 31). Native species are shaded in grey. Seeds per fruit calculated by hand from fruit collected in the Marianas unless otherwise indicated. Average seedlings per scat were calculated only from scats which contained at least one seed of that species.

		deer		pig	
species	average seeds per fruit	no. scats with this species	average seedlings per scat	no. scats with this species	average seedlings per scat
*Morinda citrifolia*	164	0	n.a.	20	55.95
*Ficus prolixa*	189	0	n.a.	3	82.33
*Carica papaya*	721	1	1	16	16.63
*Vitex parviflora*	1–2^a^	1	1	0	n.a.
*Passiflora suberosa*	26	1	8	3	4.33
*Mikania micrantha*	achene	1	1	0	n.a.
*Coccinia grandis*	126	0	n.a.	3	1
*Chromolaena odorata*	achene	0	n.a.	1	1
*Leucaena leucocephala*	18^a^	0	na	1	1
unknown		1	0.1	4	2

^a^Seeds per fruit indicated in Stone [33].

Many more seedlings emerged from the 31 pig scats that we collected from four different sites. Of these, 25 scats produced seedlings (80.6%), with a total of 1658 seedlings germinating (table 3). The eight species that germinated from pig scats included the native trees *M. citrifolia* (in 20 out of 31 scats) and *Ficus prolixa* (in three scats); the non-native trees *C. papaya* (in 16 scats) and *Leucaena leucocephala* (in one scat); the non-native vines *Passiflora suberosa* (in three scats) and *Coccinia grandis* (in three scats); and the non-native herb *Chromolaena odorata* (in one scat). All of these except for *C. odorata* and *L. leucocephala* have edible, fleshy fruits.

We used data from our vegetation surveys to compare the most abundant species that germinated from scats to the most abundant species found in nature (figure 3). Proportional abundances for species in the forest reflected their proportion of all adult trees sampled in our transects, with *Meiogyne cylindrocarpa* being the most abundant. These abundances differed dramatically from the proportional abundances in scat, suggesting some selection of these fruits by pigs in nature. That is, the species found in the highest proportion of scats for both deer and pigs did not reflect the most abundant species in forests. The most numerous seedlings germinating from scat were from many-seeded, fleshy fruited species like *Carica papaya* and *Morinda citrifolia*. While a single frugivory event could result in many seedlings, these species were found in a large proportion of the scats (proportional abundance; figure 3), indicating that these species were frequently consumed by pigs. For example, *Carica papaya* seeds germinated in 16 out of 31 (52%) pig scats. Because multiple species occurred in some single scats, the values for proportional abundance in scats do not necessarily add up to 1.

### 3.3. Effects of ungulates on community composition

**Figure 4.**
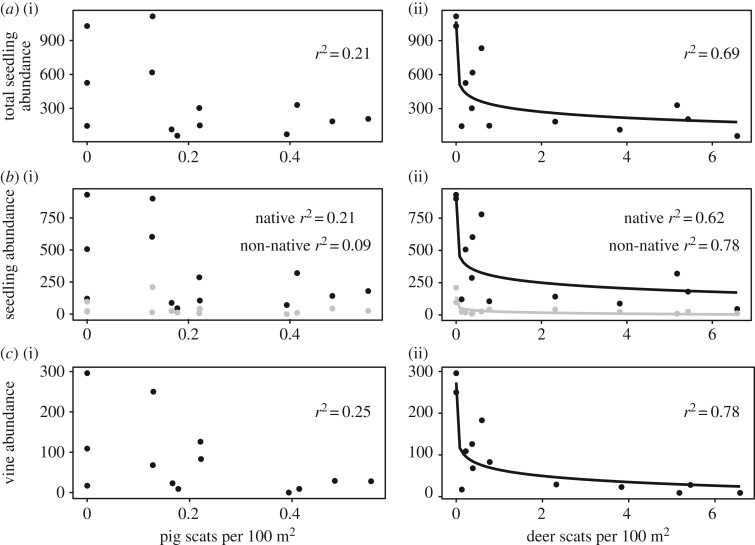
Regression analysis between pig scat abundance (*a*(i),*b*(i),*c*(i)) and deer scat abundance (*a*(ii),*b*(ii),*c*(ii)) and total seedling abundance (*a*), non-native and native seedling abundance ((*b*), with black line for native and grey line for non-native) and vine abundance (*c*). The lines in *a*(ii),*b*(ii),*c*(ii) depict a loglinear relationship.

Our surveys demonstrate a strong relationship between deer scat counts and the seedling community and fail to detect a relationship between pig scat counts and the seedling community (figure 4). A direct comparison of deer and pig effects is inappropriate because relative scat counts are species-specific; our survey methods do not allow us to obtain a *per capita* effect size. However, our surveys reflect the range of ungulate abundances found in the limestone forests of Guam. Strong negative loglinear relationships were detected between the following forest community abundances and deer scat counts: total seedling abundance (*r*^2^ = 0.77, *p* < 0.001), native seedling abundance (*r*^2^ = 0.65, *p* < 0.001), non-native seedling abundance (*r*^2^ = 0.79, *p* < 0.001) and vine abundance (*r*^2^ = 0.792, *p* < 0.001).

The two species for which seedling survival was unaffected by the ungulate exclosure treatment in the experiment above, *O. oppositifolia* and *A. marianennsis*, are also dominant in nature. After *Meiogyne cylindrocarpa*, they are the next two most common tree species for adults across all sites, accounting for over 30% of adult trees surveyed for this study (figure 3, top panel). In addition, together, they accounted for over 60% of seedlings recorded in our transects in Guam, and were present even in the areas with high deer abundance.

## 4. Discussion

We found that two non-native species often managed as a single entity—ungulates—differ in their effects at a species level. When assessing seed dispersal, we found that deer dispersed very few seeds, while pigs dispersed many seeds. The negative effects of deer were evident across the forest, as there were far more seedlings in areas with few deer than in areas with moderate or high pellet counts from deer, whereas no such correlations were detected with pig abundance (figure 4). This, in combination with the observation that most mortality in the exclosure study appeared to come from browsing rather than rooting, indicates that deer have a greater impact on seedling mortality than do pigs. While the benefits of pigs as seed dispersers were not evident in the seedling community (figure 4), neither was a negative role for pigs; herbivory by deer may mask any benefits provided via dispersal by pigs. In a novel ecosystem completely lacking native seed dispersers, the negative effects of non-native deer on seedling presence and abundance and the potential for non-native pigs to fill a missing ecological function provide support for a management approach based on functional roles rather than native/non-native status.

Both native and non-native seeds germinated from pig scats. However, fruit and seed traits are more likely than status of a plant species as native to determine whether or not pigs will disperse them. The most abundant seedlings in pig scats were from species that produce fruit containing numerous small seeds. The high number of small seeds in a given *M. citrifolia* (approx. 164 seeds/fruit)*, Ficus* sp. (approx. 189 seeds/fruit) or *C. papaya* (approx. 721 seeds/fruit) fruit means that there are many opportunities for germination once a single fruit is encountered by a pig in the forest. Both *M. citrifolia* and *C. papaya* are known to grow easily in disturbed or edge areas [38,39]. Although *C. papaya* is not a native plant, it is naturalized and not considered invasive in the Marianas [33]. It is common in Guam in previously disturbed areas and edges but not in deeper forests [31,33], indicating that it may be important for the primary or secondary succession and forest regrowth in disturbed areas. This, coupled with the results of our seedling plots indicating that both *C. papaya* and *M. citrifolia* are browsed by deer, suggests that while deer may inhibit regrowth in disturbed areas, pigs may be one of the few vertebrate species that could move successional species into edges and gaps.

Plant traits may be useful for predicting how plant species were affected by either pigs or deer. As mentioned above, *M. citrifolia, Ficus* sp. and *C. papaya* all contain a large number of small seeds per fruit, contributing to the numbers we recorded germinating from pig scats*.* They are also fleshy fruited and sweet or pungent when ripe, suggesting appeal to pigs when encountered in the forest. Small seed size may also provide a mechanical advantage that promotes dispersal over predation [40]. In terms of traits providing defence from herbivory, none of the species in our study possesses physical defences such as thorns or spikes. Studies on another species of deer suggest the chemical composition of plants affects their selection of species to browse. For example, deer tended to avoid plants with high amounts of tannic acid [41,42]. We do not have chemical composition studies on the two species that were consistently avoided in seedling exclosures (*O. oppositifolia* and *A. mariannensis*). However, *O. oppositifolia* has a thick, milky sap like other Apocynaceae, and other members of the *Aglaia* genus are known to have high tannin content [43,44], potentially contributing to lower palatability by deer. *M. citrifolia*, *P. serratifolia*, *C. papaya* and other species of *Psychotria* have documented medicinal uses [27,45–48], suggestive of potent chemical properties, but all were consumed by deer in our open seedling plots indicating that these chemical defences might not provide adequate protection from deer herbivory.

Because deer and pigs are being managed within the context of novel ecosystems, these functional differences suggest that different management strategies should apply to each species, especially in limestone karst forests. Deer are not replacing a lost ecological function, but instead have a strongly negative impact on forest communities by hindering forest regeneration (figure 4). The two most common tree seedlings across all of our survey sites and two of the most common adult species in the forests on Guam (after *Meiogyne cylindrocarpa*) are the two species that survived outside our seedling exclosures as they did inside—*O. oppositifolia* and *A. mariannensis*. This suggests that browsing preferences have already been shaping the forest species composition on Guam. Unfortunately, because both deer and pigs have been present for centuries, we had no true ‘ungulate-free’ control. To remedy this, we used exclosures and gradients of abundance to assess their effects on plant communities. Our findings are consistent with many studies that show detrimental effects of invasive deer, primarily through selective browsing [10,11,14]. We anticipate that deer eradication or control to very low abundance would prove beneficial on Guam.

While we detected negative impacts from deer, we did not detect negative impacts from pigs. Instead, pigs appear to be one of the last vertebrate seed-dispersers on an island that has lost its native dispersers. We know that pigs are present in these forests, and their wallows are abundant in secondary and ravine forests. However, the forest floor in a limestone karst forest is rocky and rigid [29], in which pigs would probably struggle to root and wallow, thereby limiting the extent of their damage to seedlings. By contrast, feral pigs in Hawaii and Malaysia cause seedling mortality, increase erosion, affect biogeochemical cycling and spread invasive plants [12,17,49,50]. These impacts are unlikely to be as severe in the rocky substrates of Guam's limestone karst forests. Rather, removing pigs from the limestone forests on Guam could have detrimental effects on plant species that have been limited by the lack of dispersers. We recognize that pigs would probably have a greater negative impact in areas, such as secondary forest or volcanic forest, with more soil. However, the role of non-native species must be evaluated on the basis of each habitat and ecological situation. While the bird-free novel ecosystems of Guam provide an important context for determining the impact of these ungulates, the limestone karst forest features are also important, because the karst is more difficult for pigs to root and wallow in than for deer to traverse and browse. A similar study in the clay soils of Guam would probably produce different results. We encourage more studies into the distribution, abundance, impacts and potential seed dispersal capabilities of pigs across all habitats on Guam.

Ungulate eradication is an important restoration tool, especially in island environments where ungulates are considered destructive invasive species [51]. Ultimately, this may be the preferred management tool for Guam's ecosystems, as efforts that have focused on removing invasive species and reintroducing native species have yielded many positive results [52–54]. However, removing invasive species could have negative consequences if these species play important ecological roles otherwise missing from the novel system [55,56]. In addition, while more research should be done on the importance of species traits in determining relationships between the plants in these ecosystems and their potential herbivores and dispersers, our results indicated that traits such as number of seeds per fruit, palatability and morphology (vine/tree/herb) were, indeed, more important than native status in determining these relationships. If restoration of native species is a future possibility, non-native species may act as a temporary placeholder until species reintroductions can occur. Restoring a functioning ecosystem rather than the exact original complement of species, or considering restoration an iterative process with strategic and temporary use of non-natives, may be more feasible for highly degraded ecosystems.

## Supplementary Material

Supplementary tables and figures for “Contrasting ecological roles of non-native ungulates in a novel ecosystem.”

## Data accessibility

Complete datasets are available at http://dx.doi.org/10.5061/dryad.sp5ff [57].
