# Supplementary material for: Contrasting ecological roles of non-native ungulates in a novel ecosystem
Source: R Soc Open Sci. 2018 Apr 25;5(4):170151. doi: 10.1098/rsos.170151 (PMC5936884; doi:10.1098/rsos.170151)
Supplement: Supplementary tables and figures for “Contrasting ecological roles of non-native ungulates in a novel ecosystem.” [file rsos170151supp1.pdf]

Supplementary information, Gawel et al. RSOS 17051

Supplementary Section 1: Sites

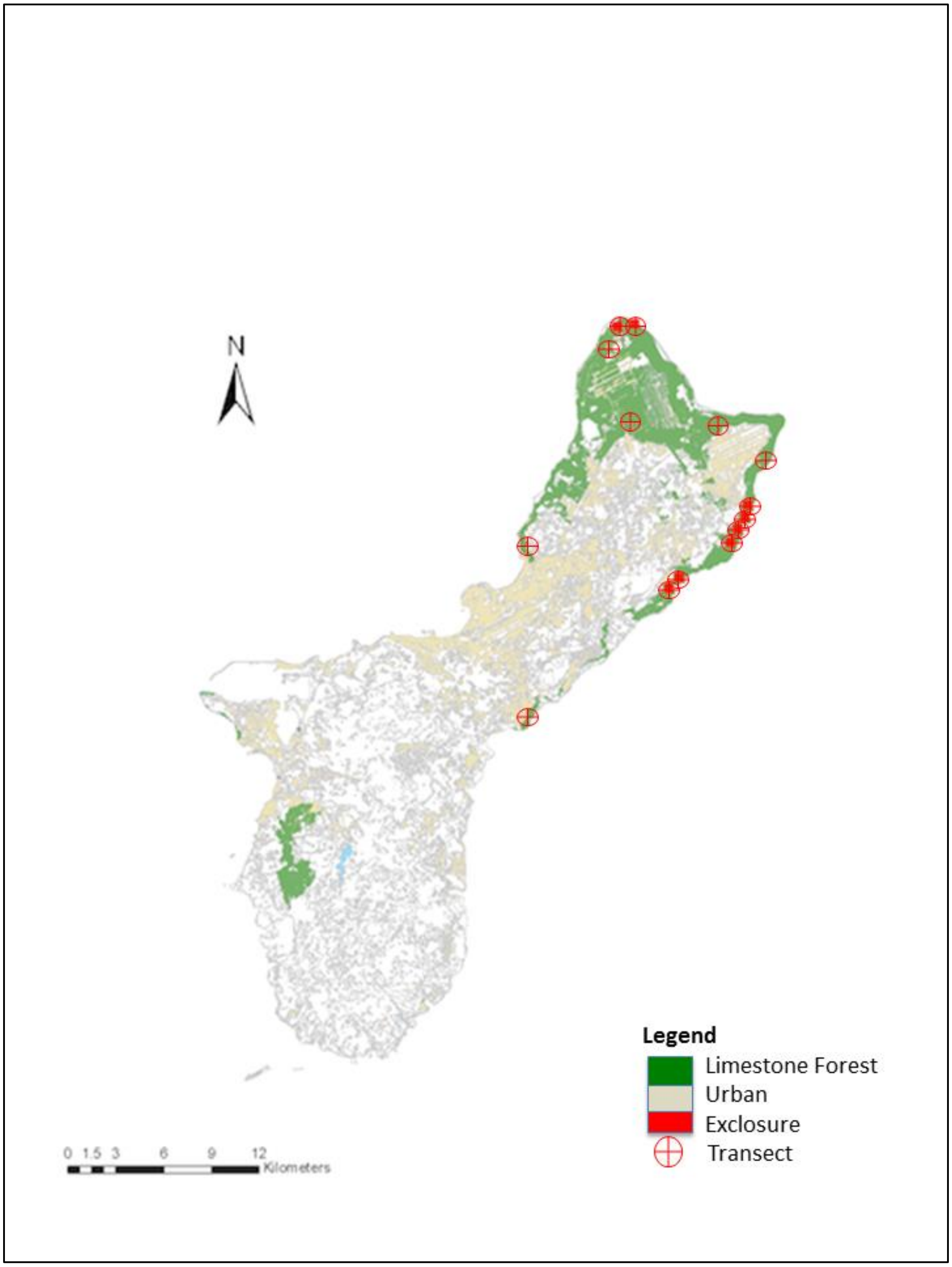

Supplementary Figure 1. Location of eight exclosures and 14 transects in Guam's limestone karst forests.

Supplementary Section 2: Plots after removing highest deer and pig density sites.

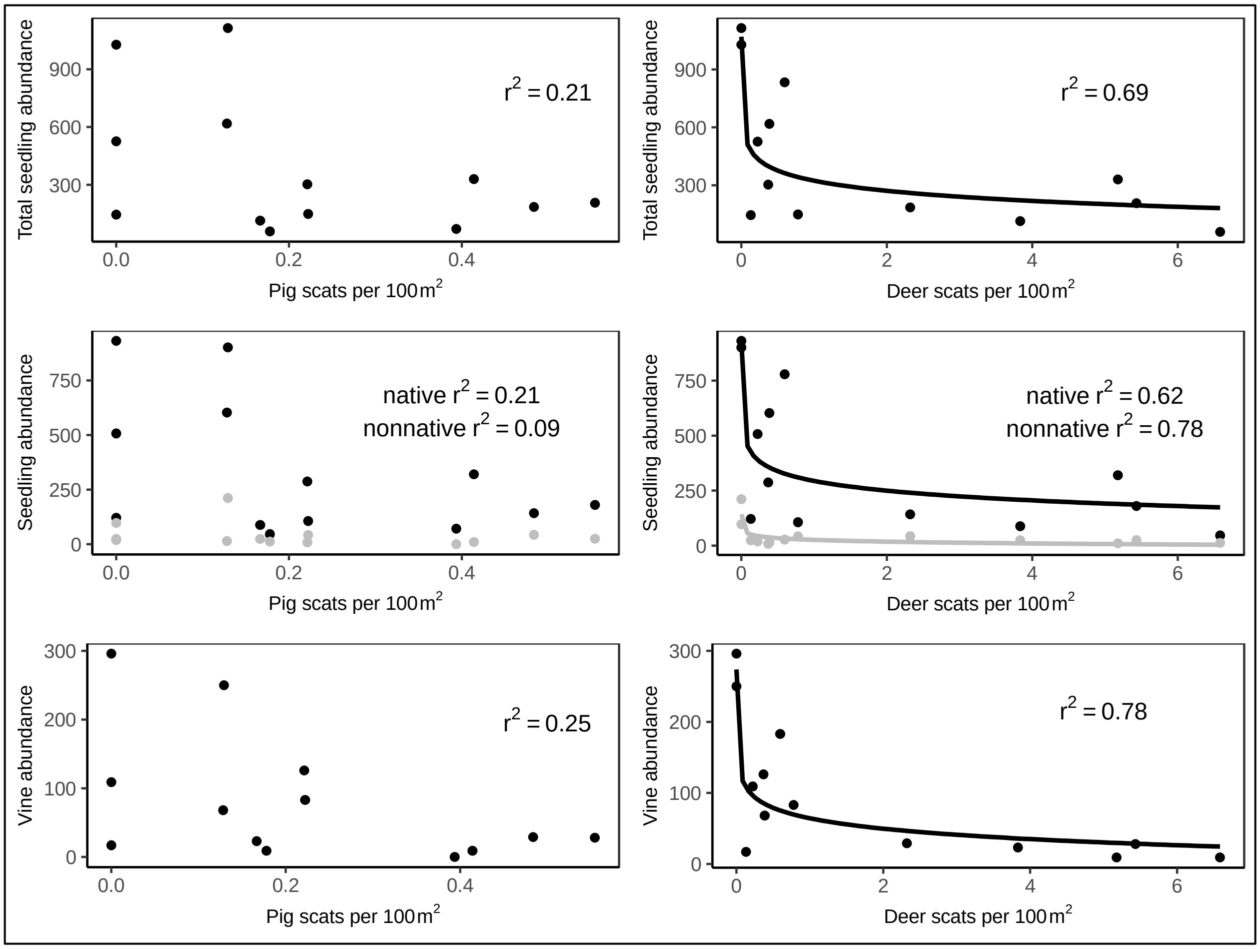

Supplementary Figure 2. Results from vegetation and scat surveys dropping site that had highest scats per $100m^2$ ("Race" for pig, and "Tartop" for deer) to determine whether the extreme value was influencing the relationship between scat count and seedling abundance. Best fit lines are only included when the fit was significant at p=0.05. A loglinear relationship was a better fit for all of the deer-seedling relationships than a linear fit. Although the scale of the x-axis is smaller, trends are qualitatively the same as those in Figure 4.

Supplementary Section 3: Comparisons of baseline characteristics of seedling plots chosen for fenced and ungulate treatments.

Supplementary Table 1: Comparison of paired plots at each site, using number of adult trees, average diameter at breast height (dbh), canopy cover, and average adult tree height.

	Fenced				Unfenced			
**Site**	**# Adult trees**	**Avg dbh (cm)**	**Canopy cover**	**Avg height (m)**	**# Adult trees**	**Avg dbh (cm)**	**Canopy cover**	**Avg height (m)**
Ritidian Grid	6	14.67	0.82	9.17	5	16.2	0.84	8.3
Ritidian Gate	7	14.57	0.9	8.71	5	11.2	0.85	7.6
Anao North	6	10	0.84	10.6	8	19	0.84	11.06
Anao South	7	12	0.73	9.43	11	6.45	0.79	9.43
North Blas	13	8.23	0.85	7.62	6	4.75	0.82	7.75
South Blas	8	6.13	0.76	6.88	6	6.67	0.74	7.67
Racetrack	14	4.93	0.9	6.21	10	4.93	0.9	6.65
Racetrack Fragment	12	4	0.88	7.46	9	4	0.89	6.67

Supplementary Table 2: We used linear mixed effects models with least square means posthoc tests to assess if the number of adult trees, proportion canopy cover, diameter at breast height of adult trees, and adult tree height differed significantly between treatments. We report output from the lsmeans tests below. We determined that these parameters do not differ significantly between seedling plots at each site, indicating plots were similar prior to the experiment.

	**Contrast values (Fenced – Unfenced)**				
**Parameter**	**Lsmean**	**Df**	**SE**	**t-ratio**	**p-value**
# adult trees	1.63	7	1.21	1.34	0.22
Canopy cover	0.00	7	0.01	0.11	0.92
Height	-0.01	127.77	0.50	-0.02	0.98
DBH	0.48	126.41	0.86	0.56	0.57

A.

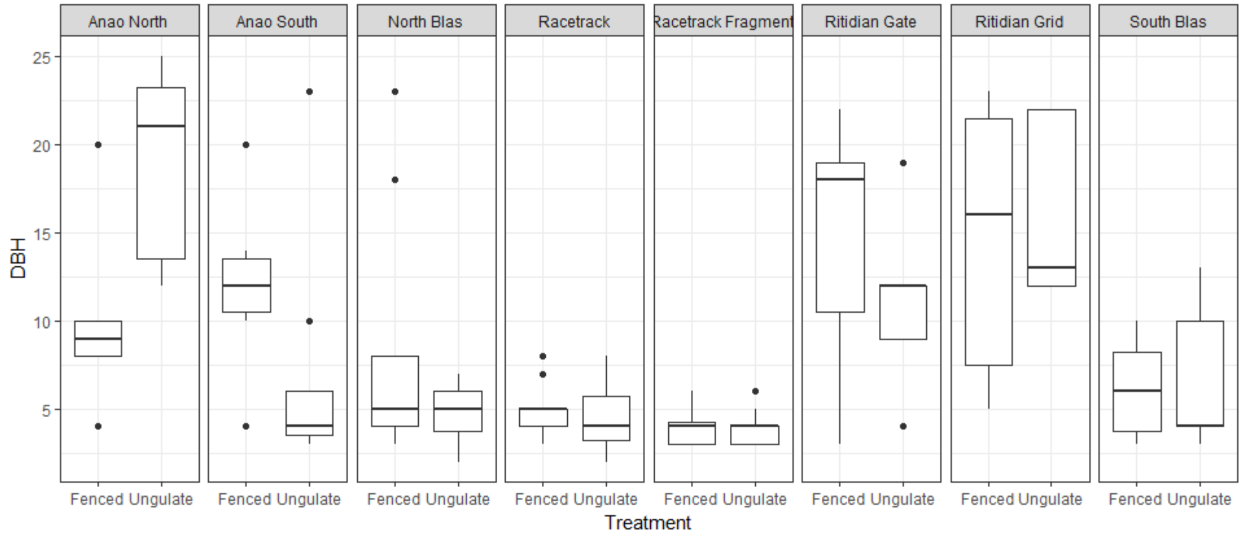

B.

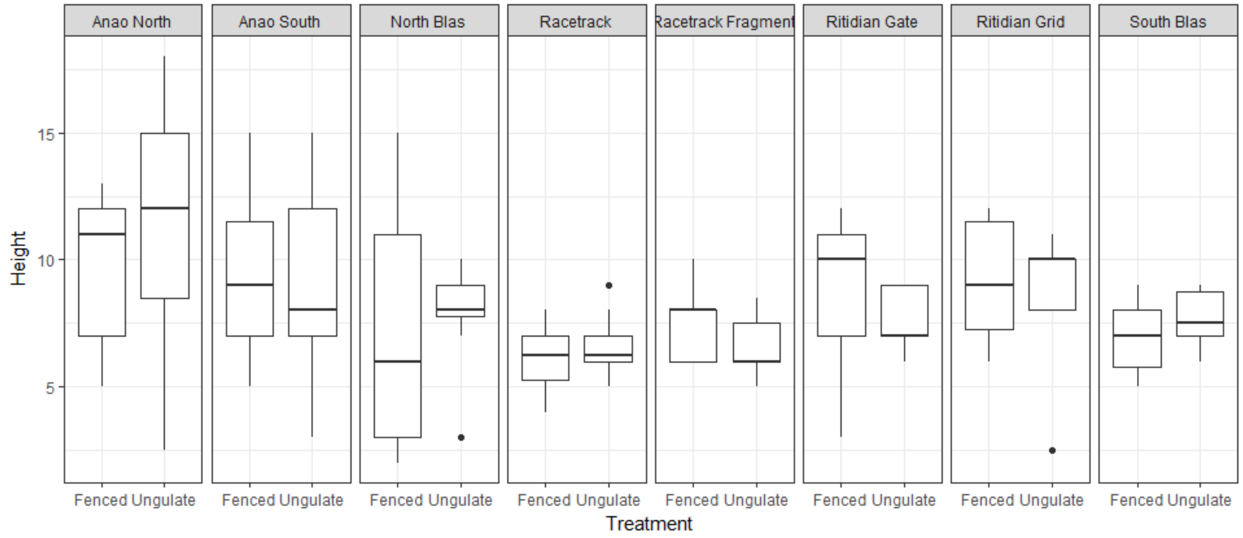

Supplementary Figure 3. Comparisons of DBH (a) and height (b) in adult trees in paired plots. While some differences are apparent, no consistent or unidirectional trends exist for either parameter across sites.

Supplementary Table 3. We included qualitative descriptions of the sites in the table below.

**Site**		**Fenced**	**Unfenced/Ungulate**
**Ritidian Grid**	**Substrate**	Solid karst formations in southeast corner of plot, large amounts of *Ochrosia oppositifolia* leaf litter	Mostly flat with scattered limestone rocks 10-20 cm in diameter, mixed leaf litter
	**Slope**	0	0
	**Species of adult trees**	*Ochrosia oppositifolia, Aglaia mariannensis, Meiogyne cylindrocarpa*	*Ochrosia oppositifolia, Psychotria mariana, Cycas micronesica*
**Ritidian Gate**	**Substrate**	Mostly 5-10cm-diameter rocks with few large, 50-cm-high boulders, <20% exposed red soil	Few large, 50-cm-high boulders, <20% exposed red soil, mostly 5-10cm-diameter rocks
	**Slope**	0	slight downward slope (<5 degrees) towards NW
	**Species of adult trees**	*Ochrosia oppositifolia, Algaia mariannensis, Triphasia trifolia*	*Aglaia mariannensis, Mammea odorata, Ochrosia oppositifolia*
**Anao North**	**Substrate**	very rocky, no soil visible, rocks from 5 - 50-cm in diameter	very rocky, no soil visible, rocks from 5 - 50-cm in diameter
	**Slope**	0	0
	**Species of adult trees**	*Ochrosia oppositifolia, Meiogyne cylindrocarpa* (*Macaranga thompsonii*, out of plot, but canopy overhanging plot)	*Ochrosia oppositifolia, Cycas micronesica, Macaranga thompsonii*
**Anao South**	**Substrate**	60% of ground cover is large rocks >20cm in diameter, some (~20% substrate) exposed red dirt	very rocky, loose rocks 10-50cm in diameter, small amounts (~10-15%) of exposed red dirt
	**Slope**	flat	slight downward slope (<5 degrees) towards east
	**# adult trees**	7	11
	**Species of adult trees**	*Mammea odorata, Aglaia mariannensis, Meiogyne cylindrocarpa, Ochrosia oppositifolia*	*Mammea odorata, Ochrosia oppositifolia, Cynometra ramiflora, Meiogyne cylindrocarpa*

Site		Fenced	Unfenced/Ungulate
**North Blas**	**Substrate**	very rocky, no soil visible, rocks approx. 50cm in diameter	very rocky, no soil visible, rocks approx. 50cm in diameter
	**Slope**	slight slope (<5 degs) downward towards NE, tower karst intermittent on all sides	in slight depression between towers of karst on north and south sides
	**Species of adult trees**	*Aglaia mariannensis, Cynometra ramiflora, Morinda citrifolia, Macaranga thompsonii, Syzigium thompsonii*	*Aglaia mariannensis, Meiogyne cylindrocarpa, Mammea odorata*
**South Blas**	**Substrate**	very rugged, large, solid rocks on south side of plot, no soil showing	rugged, no soil showing
	**Slope**	0	0
	**Species of adult trees**	*Meiogyne cylindrocarpa, Ochrosia mariannensis*	*Meiogyne cylindrocarpa, Ochrosia mariannensis, Macaranga thompsonii*
**Racetrack**	**Substrate**	moderate karst, scattered boulders 0.5-m in diameter	moderate karst, scattered boulders and rocks 0.2 to 0.5-m in diameter
	**Slope**	0	0
	**Species of adult trees**	*Eugenia reinwardtiana*	*Eugenia reinwardtiana, Meiogyne cylindrocarpa*
**Racetrack Fragment**	**Substrate**	very rocky, high amount of *Eugenia* leaf litter	very rocky, high amount of *Eugenia* leaf litter
	**Slope**	0	0
	**Species of adult trees**	*Eugenia reinwardtiana, Aglaia mariannensis*	*Eugenia reinwardtiana*

Supplementary Section 4: Model selection for linear models of vegetation characteristics explained by pig and deer scat abundance.

Supplementary Table 4. Total seedlings

	K	AICc	Delta_AICc	AICcWt
**Deer**	**3**	**206.06**	**0.00**	**0.81**
Deer + Pigs	4	209.55	3.49	0.14
Pigs	3	211.90	5.84	0.04

Supplementary Table 5. Native seedlings

	K	AICc	Delta_AICc	AICcWt
**Deer**	**3**	**202.53**	**0.00**	**0.81**
Deer + Pigs	4	205.93	3.40	0.15
Pigs	3	208.26	5.73	0.05

Supplementary Table 6. Exotic seedlings

	K	AICc	Delta_AICc	AICcWt
**Deer**	**3**	**156.83**	**0.00**	**0.72**
Pigs	3	159.64	2.81	0.18
Deer + Pigs	4	160.80	3.97	0.10

Supplementary Table 7. Vines

	K	AICc	Delta_AICc	AICcWt
**Deer**	**3**	**167.50**	**0.00**	**0.85**
Deer + Pigs	4	171.35	3.85	0.12
Pigs	3	174.59	7.09	0.02
